# Molecular Taphonomy of Heme: Chemical Degradation of Hemin under Presumed Fossilization Conditions

**DOI:** 10.3390/molecules28134887

**Published:** 2023-06-21

**Authors:** Mariam Tahoun, Marianne Engeser, Luca Svolacchia, Paul Martin Sander, Christa E. Müller

**Affiliations:** 1 PharmaCenter Bonn & Pharmaceutical Institute, Department of Pharmaceutical & Medicinal Chemistry, University of Bonn, An der Immenburg 4, 53121 Bonn, Germany; mtahoun@uni-bonn.de (M.T.); s6lusvol@uni-bonn.de (L.S.); 2 Kekulé Institute for Organic Chemistry and Biochemistry, University of Bonn, 53121 Bonn, Germany; 3 Section Paleontology, Institute of Geosciences, University of Bonn, 53115 Bonn, Germany; paulmartinsander@gmail.com

**Keywords:** heme, porphyrin, paleontology, fossilization, molecular taphonomy, preservation, mass spectrometry, dinosaurs, oxidation, hematinic acid

## Abstract

The metalloporphyrin heme acts as the oxygen-complexing prosthetic group of hemoglobin in blood. Heme has been noted to survive for many millions of years in fossils. Here, we investigate its stability and degradation under various conditions expected to occur during fossilization. Oxidative, reductive, aerobic, and anaerobic conditions were studied at neutral and alkaline pH values. Elevated temperatures were applied to accelerate degradation. High-performance liquid chromatography coupled to tandem mass spectrometry (HPLC-MS/MS) identified four main degradation products. The vinyl residues are oxidized to formyl and further to carboxylate groups. In the presence of air or H_2_O_2_, cleavage of the tetrapyrrole ring occurs, and hematinic acid is formed. The highest stability of heme was observed under anaerobic reductive conditions (half-life 9.5 days), while the lowest stability was found in the presence of H_2_O_2_ (half-life 1 min). We confirmed that the iron cation plays a crucial role in degradation, since protoporphyrin IX, lacking iron, remained significantly more stable. Under anaerobic, reductive conditions, the above-mentioned degradation products were not observed, suggesting a different degradation pathway. To our knowledge, this is the first molecular taphonomy study on heme, which will be useful for understanding its fate during fossilization.

## 1. Introduction

**Figure 1:**
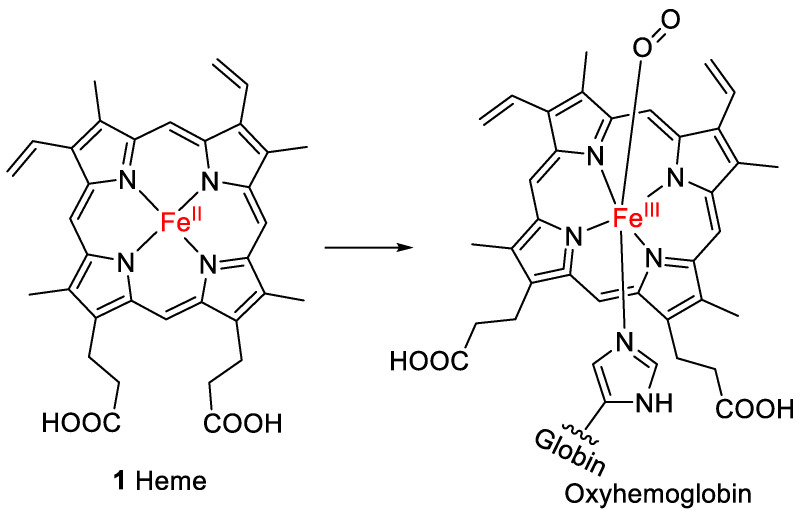
Structure of heme (**1**), the prosthetic group of oxyhemoglobin.

Heme (**1**, Figure 1) is the prosthetic group of hemoglobin, the oxygen-binding component of red blood cells in humans and all other vertebrates [1]. Heme belongs to the chemical class of metalloporphyrins, which are tetrapyrroles linked via methine bridges that coordinate a metal ion in the center of the polycyclic ring [2]. Fe^2+^ forms bonds with each of the four pyrrolic nitrogen atoms and an additional bond to the nitrogen atom of a histidine present in globin. This complex is known as deoxyhemoglobin. The Fe^2+^ can coordinate with an additional ligand, e.g., oxygen (forming oxyhemoglobin; see Figure 1), or with water or carbon monoxide [3].

**Figure 2:**
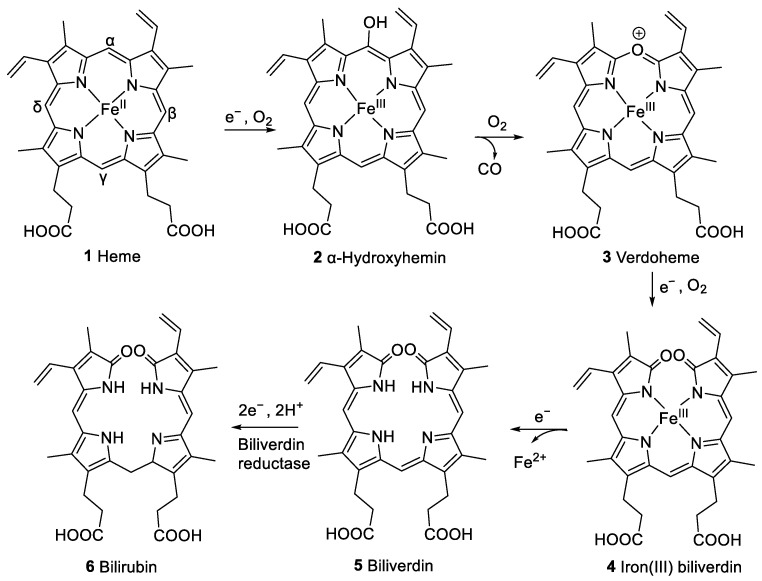
Pathway of heme (**1**) degradation by heme oxygenase to form biliverdin (**5**), including intermediate compounds **2**–**4**, and subsequent formation of bilirubin (**6**) by biliverdin reductase [4].

A previously studied pathway of heme degradation is the enzymatic degradation by heme oxygenase that occurs in vivo [4]. Heme undergoes a stepwise regiospecific oxidation of an α-methine bridge leading to ring cleavage forming α-hydroxyhemin (**2**), verdoheme (**3**), and iron(III) biliverdin (**4**) as intermediates (see Figure 2). During this reaction sequence, carbon monoxide and then iron are released subsequently, and the linear tetrapyrrole derivative biliverdin (**5**) is formed. Biliverdin reductase converts **5** to bilirubin (**6**, Figure 2), which can be conjugated with glucuronic acid to facilitate excretion. Heme oxygenase is expressed in vertebrates [5], insects, plants [6], cyanobacteria [7], algae [8], fungi [9], and bacteria [10,11,12,13,14]. This process also occurs in vitro upon the incubation of hemin, the iron(III) derivative of heme, with liver, kidney, or spleen microsomes in the presence of nicotinamide adenine dinucleotide phosphate (NADPH) [15]. This reaction is employed in coupled oxidation assays, leading to the cleavage of any of the four methine bridges and forming a mixture of four biliverdin isomers [16]. These assays serve to investigate to what extent heme is protected from free radical species in the presence of certain amounts of antioxidants, e.g., ascorbic acid [17].

**Figure 3:**
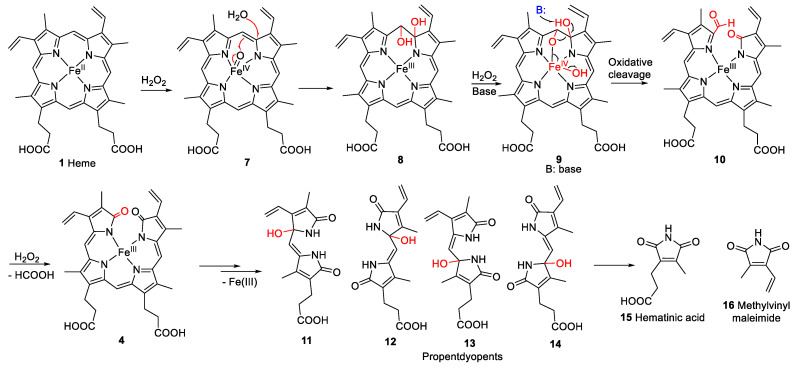
Proposed mechanism for non-enzymatic oxidative cleavage of heme (**1**) to generate monopyrrolic hematinic acid (**15**) and methylvinylmaleimide (**16**), through intermediate tetrapyrrolic (**4**, **7**–**10**) and dipyrrolic degradation products, namely the propentdyopents **11**–**14** [18,23]. The red arrows in compound **7** illustrate the rearrangement of bonds. “B” represents any base, and the black arrow illustrates the site of attack.

**Figure 4:**
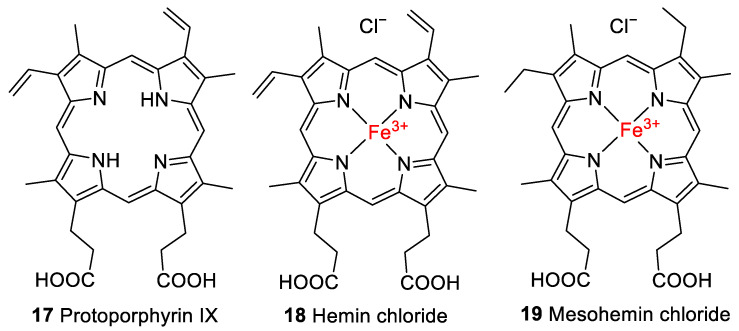
Structures of protoporphyrin IX (**17**), hemin chloride (**18**), and mesohemin chloride (**19**) employed in this study.

However, not all heme degradation pathways lead to biliverdin. NADPH-cytochrome P450 reductase degrades heme to the mono- and di-pyrrolic structures **11**–**16** via the intermediate oxidation products **7**–**10** (see Figure 3) and **4** [18,19,20]. The detailed mechanism of heme degradation by NADPH-cytochrome P450 reductase, and especially the non-enzymatic degradation of heme, e.g., under oxidative conditions, which does not lead to biliverdin, is not yet fully understood. It has been proposed that iron plays a role in the degradation by forming perferryl [iron(V)] oxygen- or oxo-iron(IV)-porphyrin π-cation radical intermediates (e.g., **7**, Figure 3) in the presence of hydrogen peroxide [18,21]. The intermediate high-valent iron complex was proposed to react with one of the methine carbon bridges forming a glycol (**8**, Figure 3). In this process, an electron is transferred, and the high-valent iron is reduced to iron(III) [22]. Another H_2_O_2_ molecule can react with the iron(III)-complex (**9**, Figure 3) leading to further cleavage of the glycol forming a compound with a *2H*-pyrrol-2-one and an α-formyl pyrrole ring (**10**, Figure 3). The α-formyl group can be hydrated and subsequently converted to an α-ketone (**4**, iron(III) biliverdin, Figure 3) after an attack by a high-valent iron oxide complex. This process takes place at each of the carbon bridges. Intermediate products are the propentdyopents, represented by a group of selected structures **11**–**14**, and the final products are the maleimides hematinic acid (**15**) and methylvinylmaleimide (**16**) and formic acid as a side product [18].

Hematinic acid has been described before as a degradation product of bilirubin (**6**) after photo-oxidation [24] and was found in the urine of newborns undergoing jaundice phototherapy [25]. It was also formed after the oxidation of Fe(III) hemin in the presence of 5% H_2_O_2_ [18] and by the oxidation of bilirubin (**6**) [26], mesoporphyrin IX [27], and chlorophyll *a* [23] with chromic acid. Hematinic acid was also described as a degradation product of hemoglobin after hemolysis with phenylhydrazine [28] and of chlorophyll *a* in senescent barley leaves [29]. Furthermore, it was detected as a bacterial degradation product of heme in *E. coli*, produced by the *E. coli* heme-utilization protein S (ChuS) [20].

Porphyrin derivatives such as heme (**1**), its iron-free derivative protoporphyrin IX (**17**, Figure 4), and biliverdin (**5**, Figure 2) have been reported to be preserved in fossils (reviewed in [2]). Preliminary evidence for the presence of heme was found in the trabecular bone of a 66-million-year-old dinosaur, *Tyrannosaurus rex* [30], by high-performance liquid chromatography (HPLC) coupled to UV/Vis detection. Heme has also been detected in 46-million-year-old mosquitoes (*Culiseta* sp.) using time-of-flight secondary-ion mass spectrometry (ToF-SIMS) [31]. A sea turtle with heme preservation in its soft tissue was preserved under similar conditions, in anaerobic fine mud at the bottom of the ancestral North Sea [32]. Protoporphyrin IX and biliverdin have been detected in 66-million-year-old fossil eggshell extracts of the oviraptorid dinosaur *Heyuannia huangi* by HPLC coupled to electrospray ionization quadrupole time-of-flight mass spectrometry [33].

Preserved organic compounds and tissue structures, e.g., blood vessels and osteocytes in dinosaurs, have become an emerging subject of interest in paleontology [34,35,36]. Organic compounds in fossils have been studied using a number of analytical methods and techniques (reviewed in [37]). To better understand the process of fossilization, experimental taphonomic studies may be performed, in which different conditions expected to have occurred during fossilization are applied to fresh material derived from living organisms [38,39,40,41]. Taphonomic studies can not only be perf–ormed on plants and animals but also on molecules. The latter approach, designated molecular taphonomy [42,43], helps to understand the possible degradation profiles of specific molecules upon fossilization.

In the present study, we performed molecular taphonomy experiments on heme under conditions that are typical for fossilization with the aim to support future analytical studies, e.g., on dinosaur bone. The employed conditions included heating, the absence or presence of air, reductive or oxidative conditions, and different pH values. Of special interest were combinations of low-oxygen or reductive conditions with mildly alkaline pH values ranging from 8 to 10, which appear to be particularly relevant for the early-stage fossilization of bone. These conditions were based on models proposed by Retallack (2001) [44] and Pfretzschner (2004) [45], who studied the relationship between pH values and oxidation/reduction potential and how this affects the preservation of fossil bones buried in soil and deposited under water, respectively. Low-oxygen conditions were likely present upon the burial of bone and inside decaying bone, even if the surroundings are oxygenated. A mildly alkaline pH value may be observed upon the dissolution of bone hydroxyapatite [45]. We are aware that the temperatures of 70 °C, 75 °C, and 95 °C used in some of our experiments are beyond what occurs during fossilization (apart from exceptional circumstances). However, heating can be expected to speed up the rate of reactions and shorten the duration of the experiment to a few days [46]. Nevertheless, the interpretation of taphonomic studies performed at high temperatures, which is a widespread approach [47], should be conducted with great care [42]. Increased temperatures may induce reactions that do not occur at lower temperatures; results might therefore not in all cases reflect the processes that happen during fossilization [42]. Another factor to consider in the interpretation of taphonomy studies is that the controlled laboratory environment is not fully realistic because the diversity of conditions that occur during fossilization may lead to incorrect interpretations of the experiments [47].

For the experiments, the iron(III) derivative of heme, known as hemin (**18**, Figure 4), with a chloride counterion was employed, which is formed by the oxidation of the iron center to the ferric form upon exposure to air. As controls, some experiments were performed with protoporphyrin IX (**17**), which lacks the iron center, or with a closely related synthetic derivative of **1**, mesohemin (iron(III) mesoporphyrin IX chloride, **19**, Figure 4), in which the vinyl residues of **1** are replaced by ethyl groups.

To monitor hemin degradation under a variety of conditions and to separate, identify, and characterize its degradation products, we used HPLC coupled to mass spectrometry and a diode array UV/Vis detector (DAD-UV/Vis), in addition to high-resolution tandem mass spectrometry (HRMS/MS). The results will be useful for understanding the degradation pathway of hemin under various conditions of fossilization.

## 2. Results and Discussion

Hemin chloride (**18**) was exposed to various conditions for different periods of time, followed by lyophilization or evaporation to dryness, dissolution in methanol or a mixture of methanol and water, and HPLC-(DAD-UV/Vis)-MS analysis. An overview of the different experimental conditions is presented in Table 1.

### 2.1. Oxidative Conditions Lead to Hematinic Acid

**Figure 5:**
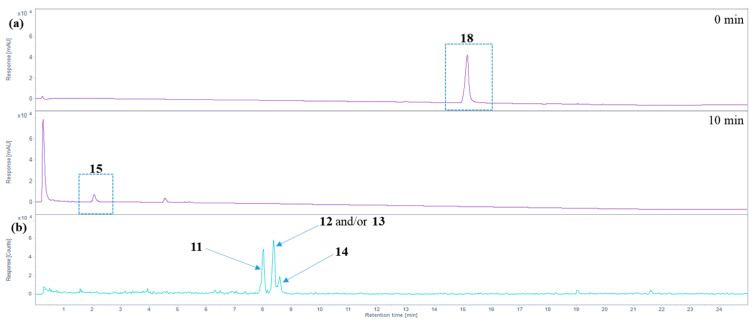
Analysis of hemin degradation. (**a**) HPLC-DAD total wavelength chromatogram before and 10 min after applying oxidizing conditions to hemin (phosphate-buffered saline, pH 7.4, 3% H_2_O_2_, RT, condition **C**, see Table 1) using HPLC-(DAD/UV)-ESI-MS. Hemin (**18**) forms hematinic acid (**15**) as the major degradation product. (**b**) Extracted ion chromatogram of *m*/*z* 319.1 ± 0.7 Da (positive ion mode), tentatively assigned to propentdyopents **11**–**14**.

The oxidative degradation of hemin chloride (**18**) in the presence of 5% H_2_O_2_ at alkaline or neutral pH values (see conditions **A**–**C** in Table 1) was rapid. Within minutes, **18** was completely degraded, and one major degradation product was detected (Figure 5a), which was found to be hematinic acid (**15**). Its structure was elucidated after isolation from the crude reaction mixture by preparative HPLC and subsequent analysis by nuclear magnetic resonance spectroscopy (NMR; see Supplementary Figures S1 and S2 for ^1^H- and ^13^C-APT-NMR spectra). Additionally, the exact mass and fragmentation pattern were confirmed by high-resolution tandem mass spectrometry: 184.06042 measured, 184.06043 calculated for [M + H]^+^; 206.04239 measured and 206.04238 calculated for [M + Na]^+^ (see Supplementary Figures S3 and S4 for mass spectra). NMR signals matched the chemical shifts reported in the literature for hematinic acid, and mass spectral data confirmed its structure [18,24,48]. Performing the experiment in methanol (in the presence of 1% NH_3_, 5% H_2_O_2_, at room temperature (RT), condition **A**, Table 1) or in water, at an alkaline pH value (1% NH_3_, pH 10.5, 5% H_2_O_2_, 60 °C for 0.5 h followed by RT, condition **B**, Table 1), or at a physiological pH value of 7.4 (phosphate-buffered saline, pH 7.4, 5% H_2_O_2_, RT, condition **C**, Table 1), had no major effect on the degradation profile.

In addition to hematinic acid (**15**), minor degradation products appeared under condition **A** (performed in methanol), the most prominent ones all showing a signal at *m*/*z* 319.1 in the positive ion mode and at *m*/*z* 317.1 in the negative mode. These mass spectra indicate the presence of molecules with a monoisotopic mass of 318.1 Da (ESI(+): [M + H]^+^ at *m*/*z* 319.1; ESI(−): [M − H]^−^ at *m*/*z* 317.1; see Figure 5b). These may be the propentdyopents **11**–**14** (reviewed in [49]), displaying a dipyrrin-1,9-dione structure (see Figure 3). As their yield was very low, no attempts were made to further characterize them beyond their mass spectra. There were three peaks for molecules with a mass of 318.1 Da, which may indicate positional isomers, of which compound **11** is possibly the first one to elute, followed by **12** and/or **13**, and finally **14**, based on previous studies on propentdyopents [23].

### 2.2. Thermal Degradation and Structural Elucidation of Intermediate Degradation Products

**Figure 6:**
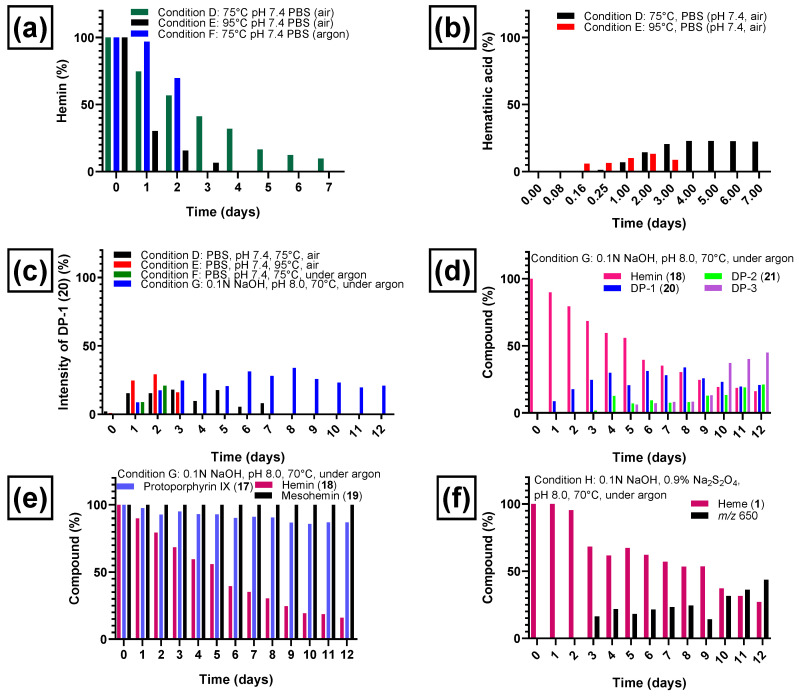
Degradation of hemin chloride (**18**) and formation of degradation products under various conditions. (**a**) Degradation of hemin chloride at pH 7.4 at elevated temperatures, in the presence or absence of air oxygen. (**b**) Formation of hematinic acid (**15**) from hemin chloride under condition **D** (phosphate-buffered saline, pH 7.4, 75 °C, air) and condition **E** (phosphate-buffered saline, pH 7.4, 95 °C, air). (**c**) Formation of degradation product DP-1 (**20**) from hemin chloride (**18**) at elevated temperatures (pH 7.4 and pH 8), in the presence or absence of air oxygen. (**d**) Degradation of hemin chloride and appearance of degradation products DP-1 (**20**), DP-2 (**21**), and DP-3 during 12 days of heating under condition **G** (in 0.1 N NaOH, pH 8, 70 °C, under argon). (**e**) Degradation profiles of protoporphyrin IX (**17**), hemin chloride (**18**), and mesohemin chloride (**19**) during 12 days of heating at 70 °C under an argon atmosphere (pH 8, condition **G**). (**f**) Degradation of heme (**1**) under condition **H** (0.1 N aq. NaOH, 0.9% sodium dithionite, pH 8, 70 °C, under argon) and formation of a major degradation product detected with mass-to-charge ratio of 650. The percentage of degradation product formation was calculated based on the ratio of its peak area to the total peak area (100%). The percentage of remaining hemin was estimated by relating its peak area to the peak area on day 0 (considered as 100% hemin).

Heating at 95 °C (see condition **E**, Table 1) at a physiological pH value of 7.4 in phosphate-buffered saline in the presence of air for several days led to a complete degradation of hemin (see Figure 6a). Hemin degradation was slower at 75 °C (see condition **D**, Table 1) as expected. The new chromatographic peaks that appeared (representing degradation products of hemin) were the same under both conditions. Hemin showed a half-life of 2.6 days at 75 °C and 0.73 days at 95 °C (Figure 6a). When the reaction was performed under argon at 75 °C (condition **F**, Table 1), hemin was found to be more stable, and 70% of the original amount remained after 2 days when the experiment was stopped. Figure 6a illustrates the degradation of hemin under conditions **D**–**F** (see Table 1 for details).

Hematinic acid (**15**, Figure 3) was the most common degradation product of hemin chloride (**18**) formed after oxidation (conditions **A**–**C**) and after heating in the presence of air (conditions **D** and **E**). A plot of the time-dependent formation of **15** under condition **D** (phosphate-buffered saline, pH 7.4, 75 °C, air) and **E** (phosphate-buffered saline, pH 7.4, 95 °C, air) is shown in Figure 6b. At 75 °C, the formation of hematinic acid (**15**) reached its maximum after 4 days. At 95 °C, the formation of **15** started earlier, but it was also further degraded, and the observed amount was lower, decreasing on the third day. The other monopyrrolic degradation product, methylvinylmaleimide (**16**), which theoretically may have been formed by heme degradation along a pathway similar to the one depicted in Figure 3, was not detected in our experiments. Hematinic acid (**15**) was not detected upon heating in the absence of oxygen under condition **F** (phosphate-buffered saline, pH 7.4, 75 °C under argon) and **G** (0.1 N NaOH, pH 8, 70 °C under argon).

Under thermal degradation conditions, we detected three other common degradation products, DP-1, DP-2, and DP-3 of initially unknown structure. DP-1, detected with a mass-to-charge ratio of 618.2, was formed in thermal degradation experiments in the presence of air (conditions **D** and **E** in Table 1) and under argon (conditions **F** and **G** in Table 1). The time-dependent formation of DP-1 under those conditions is shown in Figure 6c. In the presence of air, DP-1 reached its maximum around day 3 (18%, Figure 6c) when heated under condition **D** (phosphate-buffered saline, pH 7.4, air, 75 °C) and on day 2 (29%, Figure 6c) at 95 °C in the same medium (condition **E**). Even when heated under condition **F** (phosphate-buffered saline, pH 7.4, under argon, 75 °C), significant amounts of DP-1 were formed (Figure 6c). Thus, oxygen did not appear to be required in large amounts for its formation. The highest percentage of DP-1 was formed under weakly basic conditions (condition **G**, 0.1 N NaOH, pH 8, under argon, 70 °C), most likely due to a combination of changing the pH value, using a lower temperature, and avoiding an excess of oxygen. All of these factors may reduce the probability of further degradation of DP-1. DP-1 could not be detected after applying strongly oxidizing conditions (see conditions **A**–**C**, Table 1) due to the rapid degradation of hemin under these conditions.

**Figure 7:**
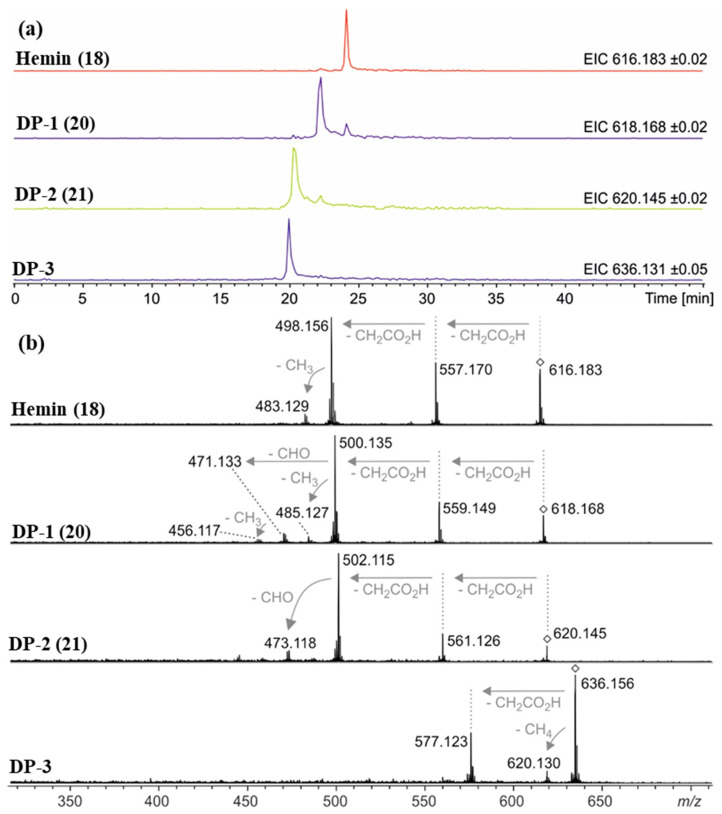
(**a**) Extracted ion chromatograms of hemin (**18**, *m*/*z* 616.2 ± 0.1 Da), degradation product 1 (DP-1, **20**, *m*/*z* 618.2 ± 0.1 Da), degradation product 2 (DP-2, **21**, *m*/*z* 620.2 ± 0.1 Da), and degradation product 3 (DP-3, *m*/*z* 636.2 ± 0.1 Da) detected via HPLC-ESI mass spectrometry after 12 days under condition G (0.1 N NaOH, pH 8, 70 °C under argon). The smaller signals approx. 2 min after the main peak for DP-1 and DP-2 are due to the isotope patterns of hemin and DP-1, respectively, as their masses fall into the mass ranges of the extracted ion chromatograms. (**b**) Collision-induced dissociation (CID) mass spectra showing the fragmentation behavior of mass-selected signals (◊) for hemin (**18**), DP-1 (**20**), DP-2 (**21**), and DP-3. In accordance with reference [50], gas-phase fragmentation of hemin proceeds via two consecutive losses of carboxymethyl radicals (∆*m* = 59.01 Da) followed by loss of methyl (∆*m* = 15.02 Da). DP-1 fragmentation starts similarly, but the third step is different: loss of CHO (∆*m* = 29.00 Da) is another indication of the formation of an aldehyde. Mass-selected ions around *m*/*z* 636.2 (DP-3) do not show loss of a CHO radical but loss of CH_4_ instead (∆*m* = 16.03 Da).

The heating of hemin under condition **G** (0.1 N NaOH, pH 8, 70 °C for 12 days under argon, Figure 6d) resulted in a slightly longer half-life (5.5 days), and more degradation products were detectable under condition **G** compared to heating condition **F**. In addition to DP-1 and many minor degradation intermediates and products, at least two other major degradation products were formed, denominated DP-2 and DP-3 according to their order of appearance (see Figure 6d and Figure 7a). Figure 6d depicts the time-dependent formation of degradation products DP-1, DP-2, and DP-3 and the parallel degradation of hemin under condition **G**. DP-1 appeared already after 1 day of heating the hemin solution at 70 °C and pH 8 under argon and reached a plateau between day 4 and day 9 (33%), followed by a steady decrease until the experiment was stopped on day 12 (21%). DP-2 was detected after 3 days and increased on day 4, when DP-1 began to reach a plateau, indicating that DP-2 could be formed from DP-1. DP-3 appeared on day 5 (Figure 6d) and steadily increased until the experiment was stopped at day 12. The minimum amount of DP-1 (21%) and the maximum amounts of DP-2 (21%) and DP-3 (45%) were thus reached on the last day of the experiment (day 12). The whole kinetic profile is in accordance with a subsequent reaction scheme in which DP-1 is formed first, followed by DP-2. DP-3 might be a subsequent reaction product of DP-1 and/or DP-2.

**Figure 8:**
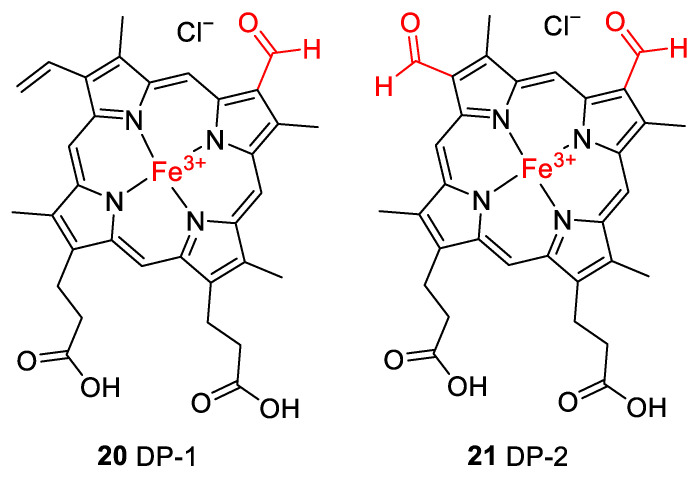
Proposed structures for hemin degradation products **20** and **21**. Note that two isomers are possible for **20** because the two inequivalent vinyl groups are oxidized with similar probability.

Accurate mass determination of the signal for DP-1 at *m*/*z* 618.1576 by high-resolution mass spectrometry (Table 2) revealed an elemental composition of C_33_H_30_N_4_O_5_Fe^+^, i.e., an oxygen atom was incorporated into the structure of hemin, and a methylene moiety was eliminated. The isotope pattern as well as the fragmentation behavior (Figure 7b) of the mass-selected ion confirmed that the iron remained complexed and the propionyl groups of hemin were still present. There was no indication that the porphyrin ring of DP-1 had not remained intact. Thus, structure **20** is proposed for the formed compound DP-1 (Figure 8), in which one of the two original vinyl groups is oxidatively degraded to an aldehyde. Note that two isomers are possible because the two vinyl groups in heme are not equivalent but probably oxidized during the degradation experiments with equal probability.

DP-2 was detected with a mass-to-charge ratio of 620.1332 in accordance with an elemental composition of C_32_H_28_N_4_O_6_Fe^+^. This result revealed that two oxygen atoms had been incorporated into the structure of hemin, and two CH_2_ moieties were eliminated (Table 2 and Figure 8). The CID gas-phase fragmentation is consistent with the presence of aldehyde groups, in addition to the two unaltered carboxyethyl side chains (Figure 7b). Thus, this corroborates the assignment of DP-2 to structure **21**. From a chemical point of view, it is plausible that both vinyl groups in heme (**1**) were successively oxidized to aldehydes yielding first DP-1 (**20**) and then DP-2 (**21**). Our determination of the structure of DP-1 and DP-2 is consistent with previous studies in which the stability of hemin used for medical purposes, e.g., to treat various porphyrias [51], was tested in solutions containing 0.1 N aq. NaOH and while studying its degradation during γ-radiotherapy [52]. However, the degradation products have only been predicted based on monitoring UV-Vis spectra [52] so far. We have now for the first time elucidated the structures of these hemin degradation products by HPLC-coupled high-resolution tandem mass spectrometry.

The recorded mass spectra for DP-3 (*m*/*z* 636.2) are not as clear as the previously discussed cases. A broadened signal is observed with an averaged mass shifting from *m*/*z* 636.159 down to 636.137 in the course of the chromatographic peak (Table 2). These findings are in accordance with a superposition of two compounds with overlapping retention times and similar masses around *m*/*z* 636.15 that are present in varying relative abundances and that unfortunately could not be separated by the limited resolving power of our Q/TOF mass spectrometer. Two matching elemental compositions are C_33_H_32_N_4_O_6_Fe^+^ (calculated *m*/*z* 636.1666) in accordance with a formal addition of H_2_O to DP-1, and C_32_H_28_N_4_O_7_Fe^+^ (calculated *m*/*z* 636.1302) indicating that three oxygen atoms were incorporated into the structure of hemin while two methylene moieties had been eliminated (Table 2). A chemically plausible putative structure for the latter ion is a structure in which one of the two aldehyde groups of **21** was oxidized to a carboxyl group. In addition to the discussed degradation products DP-1–DP-3, a series of several other minor degradation products were formed under condition **G** (in 0.1 N NaOH, pH 8, 70 °C under argon), with more than three oxygen atoms incorporated into the structure according to the detected accurate masses. This suggests that further oxidation took place, but yields were too low for structural elucidation.

### 2.3. Control Experiments Reveal Site of Degradation and Role of Iron

To further verify the involvement of the vinyl groups in hemin degradation, a control experiment under condition **G** (0.1 N NaOH, pH 8, 70 °C, under argon) was performed with mesohemin chloride, a synthetic analog of hemin chloride, in which the vinyl groups are replaced by ethyl residues (compound **19**, Figure 4). Compound **19** remained completely stable over the entire experimental period of 12 days (Figure 6e). Thus, degradation products DP-1 and DP-2 are clearly formed by the reaction of the vinyl double bond(s) in hemin by oxidative transformation to formyl groups. In accordance with the mass-spectrometric results described above, DP-1 can be assigned to **20**, ferric 2-formyl-8-vinyldeuteroporphyrin IX, and ferric 8-formyl-2-vinyldeuteroporphyrin IX, also known as *Spirographis* hemin or chlorocruorohemin (Figure 8) [53,54]. Compound **20** is found naturally as a prosthetic group of the oxygen-carrying pigment of certain species of worms from four families of marine polychaete annelids (chlorocruorin) but has not been detected in vertebrates. It was previously synthesized by the oxidation of 2-hydroxyethyl-8-vinylporphyrin [55] and protoporphyrin IX dimethyl ester [56] but has not yet been described as a degradation product of heme. Although two isomers of **20** are feasible, depending on which vinyl group was transformed into the formyl group, we only detected a single peak in the chromatogram (Figure 7) that probably represents the unresolved mixture of both isomers.

The kinetic profile of the formation of DP-2, as well as its mass-spectrometric characterization, shows that it is likely a degradation product derived from DP-1 with structure **20**. It can be concluded that DP-2 has structure **21** with two formyl groups in positions 2 and 8 of the porphyrin ring. Ferric 2,8-diformylporphyrin IX (**21**, Figure 8) has been previously synthesized [57,58]. In addition, the iron-free derivatives of **20** [59] and **21** [60] were described as photooxidation products of protoporphyrin IX (**17**). Since compounds similar to **20** and **21** could not be observed when mesohemin was studied in which reactive terminal double bonds were absent, the instability of hemin is clearly due to the presence of the vinyl groups, which is in agreement with our results as well as previous findings [61,62,63].

Among other degradation routes, one of the formyl groups in **21** may be further oxidized to a carboxylic acid yielding ferric 2-formylporphyrin IX-8-carboxylic acid as well as its positional isomer. The mass-spectrometric data give some indications for its presence in the reaction mixture of the degradation experiments. The corresponding dicarboxylic acid derivative has previously been synthesized from hemin [64].

**Figure 9:**
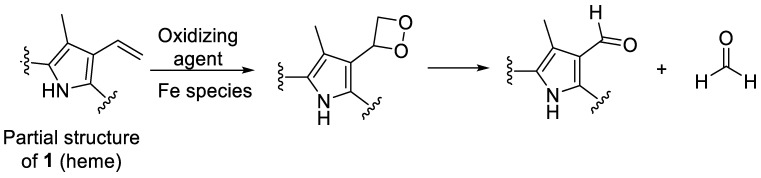
Putative mechanism for the oxidation of a vinyl group of heme to a formyl group, which is accelerated in the presence of iron [65,66].

An additional control experiment was carried out again under condition **G** (0.1 N NaOH, pH 8, 70 °C under argon) using protoporphyrin IX (**17**), the iron-free derivative of heme (Figure 4). Our aim was to investigate whether iron is involved in the oxidation reaction. Protoporphyrin IX was much more stable than hemin, with around 85% of the starting compound remaining intact after 12 days (Figure 6e). Very minor new peaks were detected in the chromatograms (amounting to less than 5% of the total peak area) at *m*/*z* 579 and 581 probably according to an incorporation of an oxygen atom and a formal addition of water, respectively. These results confirm that the iron center plays a major role in the degradation of hemin and the oxidation of its vinyl groups (see Figure 6). A putative degradation mechanism involves the addition of O_2_ to the vinyl group(s), leading to the formation of a 1,2-dioxetane, which is subsequently cleaved, generating the formyl group (Figure 9) [65]. Iron species likely catalyze this oxidative reaction [66].

### 2.4. Reductive Conditions

Finally, hemin degradation was studied under reductive conditions in the presence of sodium dithionite (condition **H**, 0.1 N NaOH and 0.9% sodium dithionite, pH 8, 70 °C, under argon). Here, iron is in the ferrous (Fe^2+^) state, and the predominant species present in solution is heme (**1**), although it is detected at *m*/*z* 616 in the electrospray mass spectra due to oxidation in the course of the ionization process. Under condition **H**, this compound was completely stable until day 2 (Figure 6f). On day 3, heme started to degrade with a half-life of about 9.5 days and was still detectable on day 12, the last day of the experiment (Figure 6f). The major degradation product was a compound with a mass-to-charge ratio of *m*/*z* 650 detected in positive mode, which appeared on day 3, increasing steadily, reaching 43% of the total peak area on day 12 (Figure 6f). We could not identify this ion so far. The elucidation of the identity of this ion is the subject of future work. Additional minor compounds were detected with mass-to-charge ratios of *m*/*z* 618 and 634, which may imply that DP-1 (**20**) was formed in small amounts followed by oxidation to the carboxylic acid derivative. DP-2 (**21**) and DP-3 were not detected under these reductive conditions. Sodium dithionite may induce a combination of oxidations and reductions, involving reactive sulfur and oxygen species. In the presence of sodium dithionite, hemin (Fe^3+^) is reduced to heme (Fe^2+^). Fe(II) is a powerful reducing agent [67,68]. Our results show that hemin is more stable under reductive conditions (half-life 9.5 days) than in the absence of reducing agents (condition **G**, 0.1 N NaOH, pH 8, 70 °C under argon, half-life 5.5 days). Reductive conditions may play a role in the early stages of fossilization and could contribute to the preservation of heme in fossils. This is consistent with the reports of heme preservation in the fossil record. The mosquitoes discussed above were embedded in an anaerobic mud at the bottom of a lake [31]. A sea turtle with heme preservation in its soft tissue was preserved under similar conditions, in anaerobic fine mud at the bottom of the ancestral North Sea [32]. In addition, the single report so far of heme in fossil bone is also plausible because of the anaerobic and alkaline microenvironment that developed quickly during decay experiments on bone [45]. We observed that in the absence of air, under slightly basic conditions of pH 8, stability was higher, while it decreased at pH 7.4. This again is consistent with decay studies on fresh bone where a pH value of 8.0–8.5 was measured [45].

## 3. Materials and Methods

The conditions used for the degradation studies on hemin are summarized in Table 1 and described in detail below.

### 3.1. Materials

Hemin chloride (product number 51280), protoporphyrin IX disodium salt (product number 258385**)**, and sodium dithionite (product number 71699) were purchased from Sigma Aldrich (Darmstadt, Germany). Ferric mesoporphyrin IX chloride (mesohemin chloride, product number sc-396889) was purchased from Santa Cruz Biotechnology Inc. (Heidelberg, Germany). Millipore water was used for LC-MS analysis (from an in-house Millipore water purification system, Darmstadt, Germany). LC-MS-grade methanol (product number 34966) was purchased from Honeywell (Offenbach/Main, Germany). Formic acid (LC-MS grade, product number 84865.180) and hydrochloric acid (37%, product number 20252.290) were purchased from VWR Chemicals (Darmstadt, Germany). Analytical-grade sodium hydroxide (product number 1375.1000) and ammonia solution (25% in water, product number 2672.1011) were purchased from Chemsolute (Renningen, Germany). Hydrogen peroxide (30% in water, product number AB129030) was purchased from abcr (Karlsruhe, Germany). Ammonium acetate (LC-MS grade, product number 73594) was purchased from Merck (Darmstadt, Germany).

### 3.2. Oxidative Degradation (Conditions **A**–**C**, Table 1)

Oxidative degradation was carried out using hydrogen peroxide in methanol containing 1% ammonia at room temperature for 10 min or in phosphate-buffered saline (pH 7.4) at room temperature for 7 h, or in water containing 1% ammonia (pH 10) at 60 °C for the initial 30 min, then continued at room temperature for up to 7 h.

#### 3.2.1. Oxidation under Alkaline Methanolic Conditions (Condition **A**, Table 1)

Hemin chloride (300 mg) was dissolved in 60 mL of methanol containing 1% ammonia, and 5 drops (0.25 mL) of aq. H_2_O_2_ were added to the solution by stirring it at room temperature. After each addition, aliquots of 10 mL were evaporated, re-suspended in 5 mL methanol, and analyzed by HPLC-(DAD-UV)-MS. At the end, a 10 mL aliquot of the remaining hemin/aq. H_2_O_2_ solution was diluted with 4 volumes of water. This solution was extracted three consecutive times with dichloromethane (50 mL for each extraction). After each extraction, the organic phase was collected. After the three extractions, the organic phases were combined, dried over magnesium sulfate, and evaporated to dryness. The residue was re-suspended in methanol to make a 1 mg/mL solution and was analyzed by HPLC-(DAD-UV)-MS. The aqueous phase remaining after the last extraction was analyzed as well.

#### 3.2.2. Oxidation under Alkaline Aqueous Conditions (Condition **B**, Table 1)

Hemin chloride (100 mg) was dissolved in 18 mL water containing 1% ammonia (pH 10.5). To this solution, 3 mL of 35% hydrogen peroxide (H_2_O_2_) solution was added (final concentration 5% H_2_O_2_ *w*/*v*). The mixture was heated for 1 h at 60 °C and then lyophilized. After resuspending the lyophilizate in methanol, the solution was diluted with 4 volumes of water and extracted three consecutive times with dichloromethane (50 mL for each extraction). After each extraction, the organic phase was collected. After the three extractions, the organic phases were combined, dried over magnesium sulfate, and evaporated to dryness. The residue was re-suspended in methanol to make a 1 mg/mL solution and was analyzed by HPLC-(DAD-UV)-MS.

#### 3.2.3. Oxidation at Physiological pH Value (Condition **C**, Table 1)

A 1.3 mg/mL (2 mM) solution of hemin chloride in phosphate-buffered saline (PBS) was set to pH 7.4 (8.55 mL of PBS, 0.45 mL of 0.1 N HCl, and 1 mL of a 20 mM hemin in 0.1 N NaOH were added). This concentration was chosen because hemin was not soluble in PBS at the high concentrations used in the previous experiments (more soluble in alkaline conditions). Portions (5 drops or 0.25 mL) of 30% *w*/*v* H_2_O_2_ were added to 5 mL of 2 mM hemin in PBS to obtain the final concentration of 5% H_2_O_2_, and the reaction was monitored for 15 min., then left to stand. Aliquots were taken for measurement with HPLC-(DAD-UV)-MS after 5, 10, and 15 min. and after left standing for 4 and 8 h.

### 3.3. Thermal Degradation

#### 3.3.1. Thermal Degradation at Physiological pH Value (Conditions **D**–**F**, Table 1)

A 1.3 mg/mL (2 mM) solution of hemin in PBS was set to pH 7.4 (8.55 mL of PBS, 0.45 mL of 0.1 N HCl, and 1 mL of a 20 mM hemin in 0.1 N NaOH were added) and heated to 75 °C (condition **D**, Table 1) or to 95 °C (condition **E**, Table 1) in a glass-stoppered flask, respectively. Aliquots were taken at 0, 2 h, 4 h, 6 h, 1 day, and then daily intervals for a total of 7 days (at 75 °C, condition **D**, Table 1) and for 3 days (95 °C, condition **E**, Table 1) and analyzed by HPLC-(DAD-UV)-MS. To study the effect of anoxic conditions, the same procedure at 75 °C was performed over 2 days under an argon atmosphere (condition **F**, Table 1). Aliquots were taken at 0, 2 h, 4 h, 6 h, 24 h, and 48 h and analyzed by HPLC-(DAD-UV)-MS.

#### 3.3.2. Thermal Degradation at Alkaline pH under an Argon Atmosphere (Condition **G**, Table 1)

A 12 mg/mL solution of hemin in 0.1 N NaOH was set to pH 8 using 0.1 N HCl and heated to 70 °C under an argon atmosphere for a total of 12 days (condition **G**, Table 1). The solution was flushed with argon at the beginning of the experiment for at least 15 min, and then the vial remained closed for 12 days. Aliquots were taken at the start and afterward daily, lyophilized and re-dissolved in methanol to give a 1 mg/mL solution, or, if not soluble in methanol, in a mixture of methanol and Millipore water, and then analyzed by HPLC-(DAD-UV)-MS. As control experiments, a 1.8 mg/mL solution of ferric mesoporphyrin IX chloride (pH 8) and a 12 mg/mL solution of protoporphyrin IX disodium salt were exposed to the same conditions for 12 days.

### 3.4. Reductive Conditions (Condition **H**, Table 1)

A 12 mg/mL solution of hemin in 0.1 N NaOH was set to pH 8 using 0.1 N HCl and heated to 70 °C in the presence of sodium dithionite (0.9% *w*/*v*) under an argon atmosphere for a total of 12 days (condition **H**, Table 1). Aliquots of 2 mL were taken at the start and daily, lyophilized and re-dissolved in methanol to give a 1 mg/mL solution, or, if not soluble in methanol, in a mixture of methanol and Millipore water, and then analyzed by HPLC-(DAD-UV)-MS.

### 3.5. Preparative Reversed-Phase HPLC for Isolation of Hematinic Acid

Isolation of hematinic acid by preparative HPLC was performed as follows: A portion of the lyophilized reaction mixture from condition A (1% NH_3_ in methanol, pH 11.5, final conc. 5% aq. H_2_O_2_, RT) having a crude weight of 3.3 g was purified by preparative reversed-phase HPLC. The method consisted of the following parameters: flow rate was 25 mL/min, mobile phase A consisted of acetonitrile + 0.05% trifluoroacetic acid (TFA), and mobile phase B consisted of water + 0.05% TFA. The run started as follows: 20% A (0–1 min), followed by a gradient that reached 100% A (1–8 min), followed by flushing with 100% A (8–16 min). This produced 41.3 mg (1.3% yield) of hematinic acid, having 93.6% purity. The structure was confirmed by high-resolution electrospray mass spectrometry (Orbitrap XL, Thermo Fisher Scientific) and by nuclear magnetic resonance spectroscopy (NMR; see Supplementary Figures S1–S4 for spectra).

### 3.6. Analysis by HPLC-(DAD-UV)-MS

Measurements were performed on an Agilent 1260 Infinity HPLC coupled to an Agilent Infinity Lab LC/MSD single-quadrupole mass spectrometer with an electrospray ion source and a DAD-UV detector (200–600 nm) (Agilent Technologies Germany GmbH & Co. KG, Waldbronn, Germany). Chromatographic separation was performed on an EC 50/3 Nucleodur C18 Gravity, 3 μm column (Macherey-Nagel, Düren, Germany) column. Mobile phase A consisted of methanol with 2 mmol/l ammonium acetate, and mobile phase B consisted of water with 0.1% formic acid. The run started with 10% A and 90% B, followed by a gradient that reached 100% of eluent A after 20 min. Then, the column was flushed for 5 min. with 100% of mobile phase A followed by 10% A and 90% B for 5 min. before starting the next run. Positive full-scan MS was obtained from 100 to 1500 *m*/*z*. The column temperature was set at 40 °C, the injection volume varied between 1 and 5 μL depending on the concentration of the sample, and the flow rate was adjusted to 0.5 mL/min. Data were collected and processed using the Data Analysis program on OpenLab CDS 2.6 software (Agilent Technologies Germany GmbH & Co. KG, Waldbronn, Germany). The extracted ion chromatogram (EIC) was used to identify known degradation products using their masses, and the DAD total wavelength chromatogram was used to calculate yields of the different degradation products at each time point. Sample concentration was approximately 1 mg/mL in methanol or a mixture of methanol and Millipore water.

### 3.7. Analysis by HPLC-Coupled High-Resolution Mass Spectrometry

Measurements were performed on a Bruker micrOTOF-Q quadrupole/time-of-flight mass spectrometer equipped with an electrospray ion source. The mass spectrometer was coupled with an Agilent HPLC 1200 Series with a UV variable wavelength detector set to 450 nm. A reversed-phase Eurospher II 100-5 C18 column (150 × 2 mm) from Knauer was used with a flow rate of 0.2 mL/min. Mobile phases A and B were used as described in Section 3.6. The run started with 10% A and 90% B for 2 min, followed by a gradient that reached 100% of eluent A after 22 min. Then, the column was flushed for 5 min with 100% of mobile phase A followed by 10% A and 90% B for 20 min before starting the next run. ESI mass spectra were recorded in positive mode in a mass range from *m*/*z* 150 to 2000 and externally calibrated with the LC-MS calibration standard Tuning Mix from Agilent. Collision-induced dissociation (CID) spectra were measured in MRM mode with a collision energy of 30–50 eV. Data were collected and processed using the Compass software package (Compass 1.3—SR1, Data Analysis 4.0—SR5, HyStar 3.2 SR4) from Bruker Daltonik GmbH, Bremen, Germany.

### 3.8. Analysis of Hematinic Acid by Nuclear Magnetic Resonance Spectroscopy (NMR)

Proton NMR (^1^H NMR) and attached proton test (APT)-carbon NMR (^13^C NMR) spectra were recorded at room temperature on a Bruker-500 spectrometer (at 500 MHz and 126 MHz, respectively) using tetramethylsilane as internal standard. Chemical shifts are reported in δ (parts per million: ppm). The following abbreviation is used for multiplicity of NMR signals: (s) singlet. For hematinic acid, the spectra were determined in (CD_3_)_2_SO (DMSO-*d*_6_): ^1^H NMR (500 MHz, DMSO-*d*_6_): *δ* 1.86 (s, 3H, CH_3_), 2.45 (s, 2H, CH_2_), 2.51 (s, 2H, CH_2_). The propionic acid and pyrrolic-NH protons were exchanged with deuterium in a D2O exchange experiment. ^13^C APT-NMR (126 MHz, DMSO-*d*_6_): *δ* 173, 31.8, 18.9, 8.3. Signals matched the chemical shifts reported in the literature for hematinic acid [18,24,48].

## 4. Conclusions

Heme, the prosthetic group of hemoglobin present in blood, was studied under a series of different, controlled conditions potentially present in the early stages of fossilization. Our aim was to simulate heme decay and identify potential degradation products formed during fossilization and preservation. The results obtained in the present study indicate that hemin, due to its central iron atom and reactive vinyl groups, has limited stability under conditions relevant for early-stage fossilization. The highest stability was observed under anaerobic reductive conditions.

Under strongly oxidative conditions, hemin is especially vulnerable and rapidly degraded. As a major degradation product of hemin, we identified hematinic acid (**15**). Under less harsh conditions, degradation of hemin is slower, and some intermediate oxidation products can be observed, i.e., ferric 2-formyl-8-vinylporphyrin IX (**20**), ferric 2,8-diformylporphyrin IX (**21**), and ferric 2-formylporphyrin IX-8-carboxylic acid, among others, including positional isomers and further oxidized products. Compounds **21** and **22** had been previously predicted but were identified now by mass spectrometry as degradation products of heme for the first time. Hematinic acid was formed only in the presence of air oxygen after heating at neutral pH or in the presence of the powerful oxidizing agent H_2_O_2_. DP-1 was formed after heating at neutral pH (7.4) or alkaline pH (8). DP-2 and DP-3 were only observed in the absence of air oxygen and at alkaline pH (8). Control experiments using mesohemin (**19**) and protoporphyrin IX (**17**) clearly showed that the vinyl groups are the site of degradation via the stepwise oxidation of the vinyl groups to formyl and further to the corresponding carboxylic acid functions and that iron plays a crucial role in these transformations. Thus, based on the findings from these experiments, the preservation of hemin in the fossil record is not unlikely, as the experiments were performed under presumed fossilization conditions, but the preservation of heme is more probable in the absence of air in a basic environment. To our knowledge, this is the first molecular taphonomy study on heme. The identification of degradation pathways and products under various conditions may be useful for further investigations on heme in fossils.

## Figures and Tables

**Table 1:** Experimental conditions employed for studying the degradation of hemin (**18**) and its observed half-life.

Condition	Medium	Concentration ^a^	Temperature	Observed Half-Life of Hemin	Identified Degradation Product(s)
Oxidation under alkaline conditions					
**A**	1% NH_3_ in methanol + aq. H_2_O_2_ (final conc. 5%)	5 mg/mL hemin	Room temperature	≤1 min	Hematinic acid (**15**)
**B**	1% NH_3_ in water (pH 10.5) + aq. H_2_O_2_ (final conc. 5%)	5.5 mg/mL hemin	60 °C for the initial 30 min, then room temperature	≤1 min	Hematinic acid (**15**)
Oxidation at physiological pH value					
**C**	Phosphate-buffered saline (PBS pH 7.4) + aq. H_2_O_2_ (final conc. 3%)	1.3 mg/mL hemin	Room temperature	≤1 min	Hematinic acid (**15**)
Heating at physiological pH value					
**D**	PBS (pH 7.4) in the presence of air	1.3 mg/mL hemin	75 °C	2.6 days	Hematinic acid (**15**) DP-1 (**20**)
**E**	PBS (pH 7.4) in the presence of air	1.3 mg/mL hemin	95 °C	0.73 days	Hematinic acid (**15**) DP-1 (**20**)
**F**	PBS (pH 7.4) under an argon atmosphere	1.3 mg/mL hemin	75 °C	n.d. ^b^	Hematinic acid (**15**) DP-1 (**20**)
Heating under alkaline conditions					
**G**	0.1N aq. NaOH (pH 8) under an argon atmosphere (control experiments using compounds 18 and 20)	12 mg/mL hemin, 1.8 mg/mL ferric mesoporphyrin IX (19), or 12 mg/mL protoporphyrin IX (17)	70 °C	5.5 days (17: minor degradation; 19: no degradation)	DP-1 (**20**) DP-2 (**21**) DP-3
**H**	0.1 N aq. NaOH (pH 8) + 0.9% Na_2_S_2_O_4_ under an argon atmosphere	12 mg/mL hemin		9.5 days	Unknown degradation product (mass of 650 Da)

^a^ The highest concentration possible was used to identify as many degradation products as possible. Half-lives were determined for each condition from the curve showing exponential degradation. ^b^ The half-life could not be predicted since the experiment was stopped after 2 days before 50% of hemin degradation had occurred. Note that the conditions **A**–**H** are given in bold in order to facilitate identifying them.

**Table 2:** Mass-spectrometric data of hemin and its degradation products determined by high-resolution mass spectrometry (HRMS).

Compound ^a^	Molecular Ion (Calculated Mass)	Molecular Formula	Accurate Mass Determination (HRMS)
Hemin (**18**)	616.1768	C_34_H_32_N_4_O_4_Fe	616.1785
DP-1 (**20**)	618.1561	C_33_H_30_N_4_O_5_Fe	618.1576
DP-2 (**21**)	620.1353	C_32_H_28_N_4_O_6_Fe	620.1332
DP-3	636.1302 636.1666	C_32_H_28_N_4_O_7_Fe or C_33_H_32_N_4_O_6_Fe	636.137–636.159

^a^ DP, degradation product.

## Data Availability

All data are contained within this article or its Supplementary Materials or are available upon reasonable request.

## Supplementary Materials

The following Supporting Information can be downloaded at https://www.mdpi.com/article/10.3390/molecules28134887/s1: Figure S1: ^1^H-NMR spectrum of the isolated hematinic acid, including signal assignments, determined in DMSO-*d*_6_, Figure S2: ^13^C attached proton test (APT) NMR spectrum of the isolated hematinic acid, including signal assignments, determined in DMSO-*d*_6_, Figure S3: Orbitrap XL high-resolution mass spectra of the isolated hematinic acid, showing its molecular ions at *m*/*z* 184.06042 (calculated 184.06043 for [M + H]^+^) and *m*/*z* 206.04239 (206.04238 calculated for [M + Na]^+^), Figure S4: High-resolution collision-induced dissociation mass spectra of the isolated hematinic acid, showing its fragmentation pattern to the major fragments 166.051, 138.056, and 84.045 *m*/*z*.

## Sample Availability

Samples of the isolated compound hematinic acid are available from the authors upon reasonable request while supplies last.

## References

[1] Giardina B., Messana I., Scatena R., Castagnola M. (1995). The multiple functions of hemoglobin. Crit. Rev. Biochem. Mol. Biol..

[2] Tahoun M., Gee C.T., McCoy V.E., Sander P.M., Müller C.E. (2021). Chemistry of porphyrins in fossil plants and animals. RSC Adv..

[3] Kundu S., Trent J.T., Hargrove M.S. (2003). Plants, humans and hemoglobins. Trends Plant Sci..

[4] Yoshida T., Migita C.T. (2000). Mechanism of heme degradation by heme oxygenase. J. Inorg. Biochem..

[5] Kikuchi G., Yoshida T., Noguchi M. (2005). Heme oxygenase and heme degradation. Biochem. Biophys. Res. Commun..

[6] Terry M.J., Linley P.J., Kohchi T. (2002). Making light of it: The role of plant haem oxygenases in phytochrome chromophore synthesis. Biochem. Soc. Trans..

[7] Cornejo J., Beale S.I. (1997). Phycobilin biosynthetic reactions in extracts of cyanobacteria. Photosynth. Res..

[8] Beale S.I. (1993). Biosynthesis of phycobilins. Chem. Rev..

[9] Pendrak M.L., Chao M.P., Yan S.S., Roberts D.D. (2004). Heme oxygenase in *Candida albicans* is regulated by hemoglobin and is necessary for metabolism of exogenous heme and hemoglobin to α-biliverdin. J. Biol. Chem..

[10] Schmitt M.P. (1997). Utilization of host iron sources by *Corynebacterium diphtheriae*: Identification of a gene whose product is homologous to eukaryotic heme oxygenases and is required for acquisition of iron from heme and hemoglobin. J. Bacteriol..

[11] Wilks A., Schmitt M.P. (1998). Expression and characterization of a heme oxygenase (Hmu O) from *Corynebacterium diphtheriae*. J. Biol. Chem..

[12] Melanie R., Wenming Z., Rahul D., Angela W., Igor S. (2001). Homologues of neisserial heme oxygenase in Gram-negative bacteria: Degradation of heme by the product of the *pigA* gene of *Pseudomonas aeruginosa*. J. Bacteriol..

[13] Zhu W., Wilks A., Stojiljkovic I. (2000). Degradation of heme in Gram-negative bacteria: The product of the *hemO* gene of neisseriae is a heme oxygenase. J. Bacteriol..

[14] Zhu W., Hunt D.J., Richardson A.R., Stojiljkovic I. (2000). Use of heme compounds as iron sources by pathogenic neisseriae requires the product of the *hemO* gene. J. Bacteriol..

[15] Tenhunen R., Marver H.S., Schmid R. (1968). The enzymatic conversion of heme to bilirubin by microsomal heme oxygenase. Proc. Natl. Acad. Sci. USA.

[16] Bonnett R., McDonagh A.F. (1973). The meso-reactivity of porphyrins and related compounds. Part VI. Oxidative cleavage of the haem system. The four isomeric biliverdins of the IX series. J. Chem. Soc. Perkin Trans. 1.

[17] Giulivi C., Cadenas E. (1993). The reaction of ascorbic acid with different heme iron redox states of myoglobin. FEBS Lett..

[18] Schaefer W.H., Harris T.M., Guengerich F.P. (1985). Characterization of the enzymic and nonenzymic peroxidative degradation of iron porphyrins and cytochrome P-450 heme. Biochemistry.

[19] Guengerich F.P. (1978). Destruction of heme and hemoproteins mediated by liver microsomal reduced nicotinamide adenine dinucleotide phosphate-cytochrome P-450 reductase. Biochemistry.

[20] Ouellet Y.H., Ndiaye C.T., Gagné S.M., Sebilo A., Suits M.D.L., Jubinville É., Jia Z., Ivancich A., Couture M. (2016). An alternative reaction for heme degradation catalyzed by the *Escherichia coli* O157:H7 ChuS protein: Release of hematinic acid, tripyrrole and Fe(III). J. Inorg. Biochem..

[21] Groves J.T., Haushalter R.C., Nakamura M., Nemo T.E., Evans B.J. (1981). High-valent iron-porphyrin complexes related to peroxidase and cytochrome P-450. J. Am. Chem. Soc..

[22] Takahashi A., Kurahashi T., Fujii H. (2011). Redox potentials of oxoiron(IV) porphyrin π-cation radical complexes: Participation of electron transfer process in oxygenation reactions. Inorg. Chem..

[23] Ritter M., Oetama V.S.P., Schulze D., Muetzlaff K., Meents A.K., Seidel R.A., Görls H., Westerhausen M., Boland W., Pohnert G. (2020). Pyrrolic and dipyrrolic chlorophyll degradation products in plants and herbivores. Chem.-Eur. J..

[24] Lightner D.A., Quistad G.B. (1972). Hematinic acid and propentdyopents from bilirubin photo-oxidation in vitro. FEBS Lett..

[25] Lightner D.A., Linnane W.P., Ahlfors C.E. (1984). Bilirubin photooxidation products in the urine of jaundiced neonates receiving phototherapy. Pediatr. Res..

[26] Rüdiger W. (1970). Recent chemistry and biochemistry of bile pigments. Angew. Chem. Int. Ed. Engl..

[27] Muir H.M., Neuberger A. (1950). The biogenesis of porphyrins. 2. The origin of the methyne carbon atoms. Biochem. J..

[28] Hirota K., Sasaki K. (1994). Production of hematinic acid by the reaction of hemoglobin with phenylhydrazine: Evidence for the oxidative cleavage of heme. Biol. Pharm. Bull..

[29] Suzuki Y., Shioi Y. (1999). Detection of chlorophyll breakdown products in the senescent leaves of higher plants. Plant Cell Physiol..

[30] Schweitzer M.H., Marshall M., Carron K., Bohle D.S., Busse S.C., Arnold E.V., Barnard D., Horner J.R., Starkey J.R. (1997). Heme compounds in dinosaur trabecular bone. Proc. Natl. Acad. Sci. USA.

[31] Greenwalt D.E., Goreva Y.S., Siljeström S.M., Rose T., Harbach R.E. (2013). Hemoglobin-derived porphyrins preserved in a Middle Eocene blood-engorged mosquito. Proc. Natl. Acad. Sci. USA.

[32] Lindgren J., Kuriyama T., Madsen H., Sjövall P., Zheng W., Uvdal P., Engdahl A., Moyer A.E., Gren J.A., Kamezaki N. (2017). Biochemistry and adaptive colouration of an exceptionally preserved juvenile fossil sea turtle. Sci. Rep..

[33] Wiemann J., Yang T.R., Sander P.N., Schneider M., Engeser M., Kath-Schorr S., Müller C.E., Sander P.M. (2017). Dinosaur origin of egg color: Oviraptors laid blue-green eggs. PeerJ.

[34] Wiersma K., Läbe S., Sander P.M., Gee C.T., McCoy V.E., Sander P.M. (2021). Organic phase preservation in fossil dinosaur and other tetrapod bone from deep time. Fossilization: Understanding the Material Nature of Ancient Plants and Animals.

[35] Schweitzer M.H., Wittmeyer J.L., Horner J.R. (2007). Soft tissue and cellular preservation in vertebrate skeletal elements from the Cretaceous to the present. Proc. R. Soc. B Biol. Sci..

[36] Schweitzer M.H. (2011). Soft tissue preservation in terrestrial Mesozoic vertebrates. Annu. Rev. Earth Planet. Sci..

[37] Tahoun M., Engeser M., Namasivayam V., Sander P.M., Müller C.E. (2022). Chemistry and analysis of organic compounds in dinosaurs. Biology.

[38] Behrensmeyer A.K., Kidwell S.M. (1985). Taphonomy’s contributions to paleobiology. Paleobiology.

[39] Behrensmeyer A.K., Kidwell S.M., Gastaldo R.A. (2000). Taphonomy and paleobiology. Paleobiology.

[40] Gifford D.P. (1981). Taphonomy and paleoecology: A critical review of archaeology’s sister disciplines. Advances in Archaeological Method and Theory.

[41] Schweitzer M.H., Zheng W., Cleland T.P., Goodwin M.B., Boatman E., Theil E., Marcus M.A., Fakra S.C. (2014). A role for iron and oxygen chemistry in preserving soft tissues, cells and molecules from deep time. Proc. R. Soc. B Biol. Sci..

[42] Eglinton G., Logan G.A. (1991). Molecular preservation. Philos. Trans. R. Soc. Lond. B Biol. Sci..

[43] Latham K.E., Miller J.J. (2019). DNA recovery and analysis from skeletal material in modern forensic contexts. Forensic Sci. Res..

[44] Retallack G.J. (2001). Organisms. Soils of the Past: An Introduction to Paleopedology.

[45] Pfretzschner H.-U. (2004). Fossilization of Haversian bone in aquatic environments. Comptes Rendus Palevol.

[46] Von Endt D.W., Ortner D.J. (1984). Experimental effects of bone size and temperature on bone diagenesis. J. Archaeol. Sci..

[47] Briggs D.E.G., McMahon S. (2016). The role of experiments in investigating the taphonomy of exceptional preservation. Palaeontology.

[48] Brynjelsen S.E., Doty M., Poss M.J. (1998). Facile synthesis of hematinic acid. Synth. Commun..

[49] Tomat E. (2019). Propentdyopents: Brief history of a family of dipyrrolic pigments. J. Porphyr. Phthalocyanines.

[50] Charkin O.P., Klimenko N.M., Nguyen P.T., Charkin D.O., Mebel A.M., Lin S.H., Wang Y.-S., Wei S.-C., Chang H.-C. (2005). Fragmentation of heme and hemin+ with sequential loss of carboxymethyl groups: A DFT and mass-spectrometry study. Chem. Phys. Lett..

[51] Siegert S.W.K., Holt R.J. (2008). Physicochemical properties, pharmacokinetics, and pharmacodynamics of intravenous hematin: A literature review. Adv. Ther..

[52] Rothschild M.-L., Cosi L., Myers L.S. (1958). Effect of gamma-radiation on ferriprotoporphyrin. Nature.

[53] Jackson A.H., Kenner G.W., Wass J. (1974). Pyrroles and related compounds. Part XXV. Pemptoporphyrin, isopemptoporphyrin, and chlorocruoroporphyrin (*Spirographis* porphyrin). J. Chem. Soc. Perkin Trans. 1.

[54] Inhoffen H.H., Bliesener C., Brockmann H. (1966). Zur weiteren Kenntnis des Chlorophylls und des Hämins, VIII.: Umwandlung von Protoporphyrin IX über Photoprotoporphyrin in Spirographis- und Isospirographisporphyrin. Tetrahedron Lett..

[55] Fischer H., Wecker G. (1942). Synthese des Spirographisporphyrins. Hoppe. Seylers. Z. Physiol. Chem..

[56] Fischer H., Deilmann K.-O. (1944). Überführung von Hämin IX in Spirographisporphyrin und über einige Derivate des Deuteroporphyrins. Hoppe. Seylers. Z. Physiol. Chem..

[57] Tsubaki M., Nagai K., Kitagawa T. (1980). Resonance Raman spectra of myoglobins reconstituted with spirographis and isospirographis hemes and iron 2, 4-diformylprotoporphyrin. Biochemistry.

[58] Sono M., Asakura T. (1974). Separation and properties of spirographis and isospirographis porphyrin dimethyl esters. Biochemistry.

[59] Inhoffen H.H., Brockmann H., Bliesener K.-M. (1969). Zur weiteren Kenntnis des Chlorophylls und des Hämins, XXX Photoprotoporphyrine und ihre Umwandlung in Spirographis-sowie Isospirographis-porphyrin). Justus Liebigs Ann. Chem..

[60] Horsey B.E., Whitten D.G. (1978). Photochemical reactions in organized monolayer assemblies. 8. Environmental effects on photochemical reactions: Contrasts in the photooxidation behavior of protoporphyrin IX in solution, monolayer films, organized monolayer assemblies, and micelles. J. Am. Chem. Soc..

[61] Drabkin D.L. (1942). Spectrophotometric studies: X. Structural interpretation of the spectra of cyanide, pyridine, and carbon monoxide derivatives of cytochrome *c* and hemoglobin. J. Biol. Chem..

[62] Rothschild M.-L., Myers L.S. (1958). The spontaneous change of ferriprotoporphyrin in alkaline solution. Nature.

[63] Rothschild M.-L. (1960). The reaction of ferriprotoporphyrin with hydrogen peroxide in alkaline solutions. Arch. Biochem. Biophys..

[64] Fischer H., Deilmann K.-O. (1940). Überführung von Hämin in Deuteroporphyrin-2,4-dicarbonsäure-tetramethylester und von Hämatoporphyrin in Diacetyl-deuteroporphyrin. Justus Liebigs Ann. Chem..

[65] Cox G.S., Whitten D.G. (1982). Mechanisms for the photooxidation of protoporphyrin IX in solution. J. Am. Chem. Soc..

[66] Gonzalez-de-Castro A., Xiao J. (2015). Green and efficient: Iron-catalyzed selective oxidation of olefins to carbonyls with O_2_. J. Am. Chem. Soc..

[67] Li X., Liu T., Li F., Zhang W., Zhou S., Li Y. (2012). Reduction of structural Fe(III) in oxyhydroxides by *Shewanella decolorationis* S12 and characterization of the surface properties of iron minerals. J. Soils Sediments.

[68] Yan B., Wrenn B.A., Basak S., Biswas P., Giammar D.E. (2008). Microbial reduction of Fe(III) in hematite nanoparticles by *Geobacter sulfurreducens*. Environ. Sci. Technol..

