# Supplementary material for: Molecular Taphonomy of Heme: Chemical Degradation of Hemin under Presumed Fossilization Conditions
Source: Molecules. 2023 Jun 21;28(13):4887. doi: 10.3390/molecules28134887 (PMC10343206; doi:10.3390/molecules28134887)
Supplement: Supplementary file 1 [file molecules-28-04887-s001.zip › molecules-2445807-supplementary.pdf]

Supporting Information

Molecular taphonomy of heme: Chemical degradation of hemin under presumed fossilization conditions

Mariam Tahoun [1], Marianne Engeser [2]*, Luca Svolacchia [1], Paul Martin Sander [3] and Christa E. Müller [1,]*

[1] PharmaCenter Bonn & Pharmaceutical Institute, Department of Pharmaceutical and Medicinal Chemistry, University of Bonn, An der Immenburg 4, D-53121 Bonn, Germany; mtahoun@uni-bonn.de; s6lusvol@uni-bonn.de; christa.mueller@uni-bonn.de

[2] Kekulé Institute for Organic Chemistry and Biochemistry, University of Bonn, D-53121 Bonn, Germany; marianne.engeser@uni-bonn.de

[3] Institute of Geosciences, Department of Paleontology, University of Bonn, D-53113 Bonn, Germany; paulmartinsander@gmail.com

***** Correspondence: christa.mueller@uni-bonn.de; marianne.engeser@uni-bonn.de

Table of Contents

I. ^{1}H-NMR and ^{13}C-NMR spectra of isolated hematinic acid 2

II. High-resolution mass spectra of isolated hematinic acid 3

I. ^{1}H-NMR and ^{13}C-NMR spectra of isolated hematinic acid

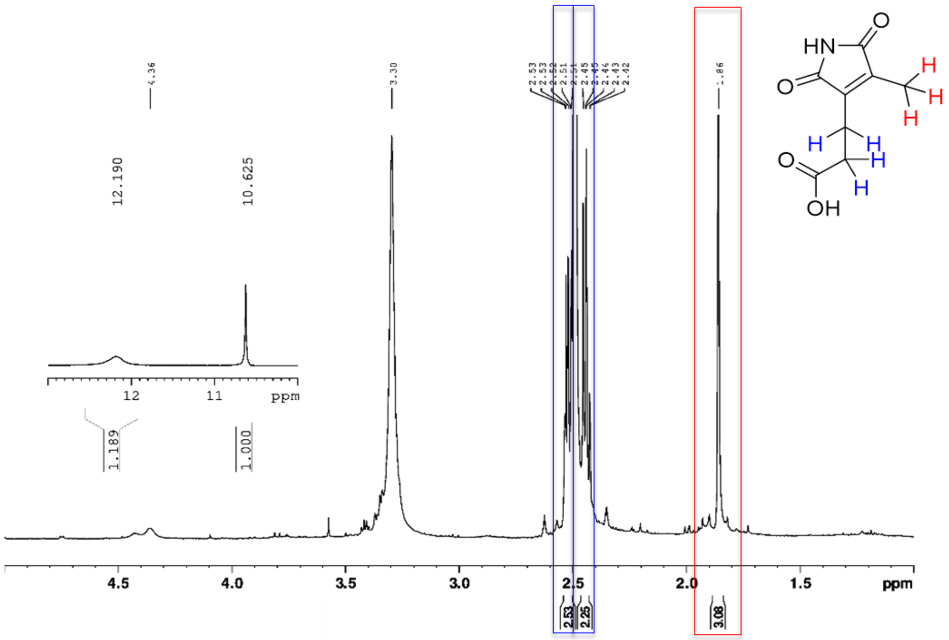

Figure S1. ^{1}H NMR spectrum of isolated hematinic acid determined in DMSO-d_6.

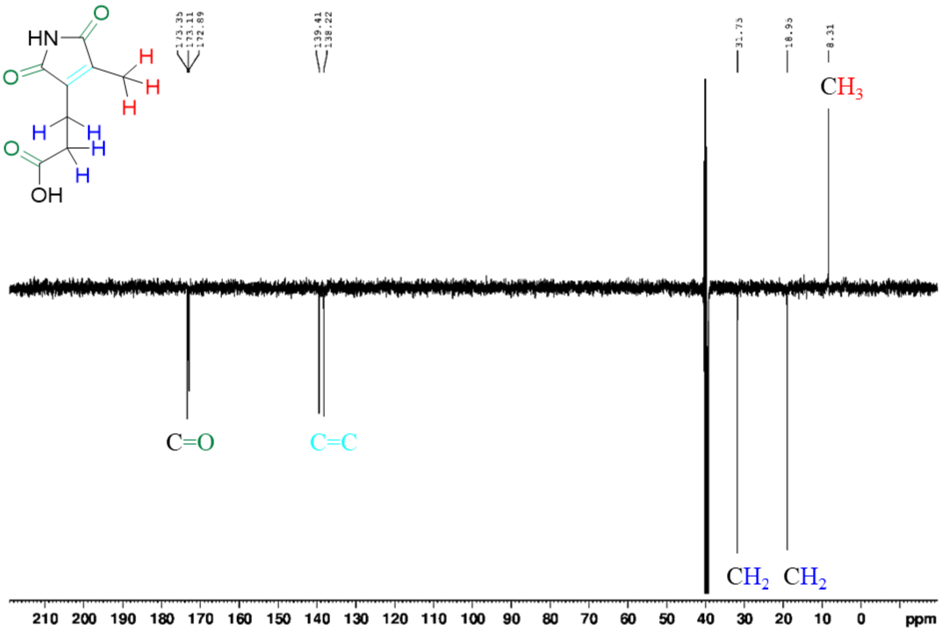

Figure S2. ^{13}C NMR spectrum (attached proton test, APT) of isolated hematinic acid determined in DMSO-d_6.

II. High-resolution mass spectra of isolated hematinic acid

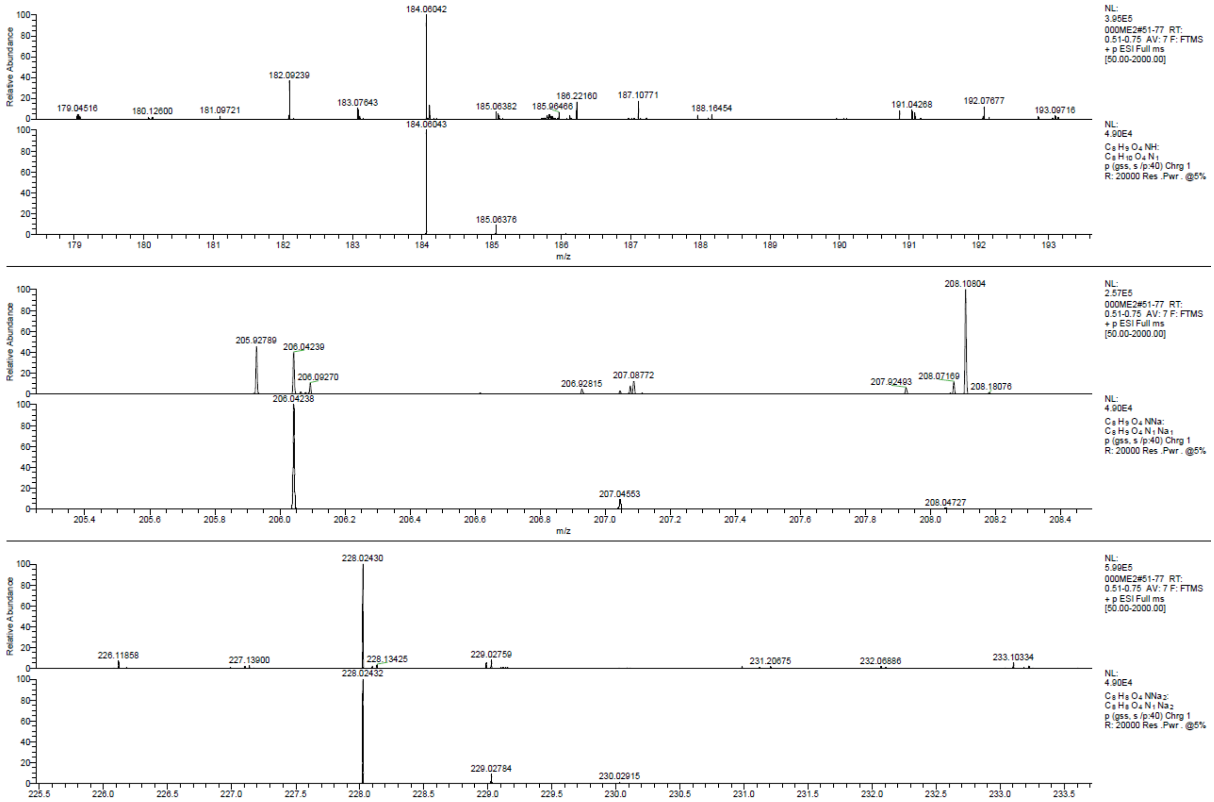

Figure S3. High resolution direct infusion electrospray mass spectra (Orbitrap XL) of isolated hematinic acid showing its molecular ions $[M+H]^+$ at *m/z* 184.06043 (top), $[M+Na]^+$ at *m/z* 206.04239 (top), and $[M+2Na-H]^+$ at *m/z* 228.02430 (bottom). In each case, the measured spectrum is shown above, the calculated one below for comparison.

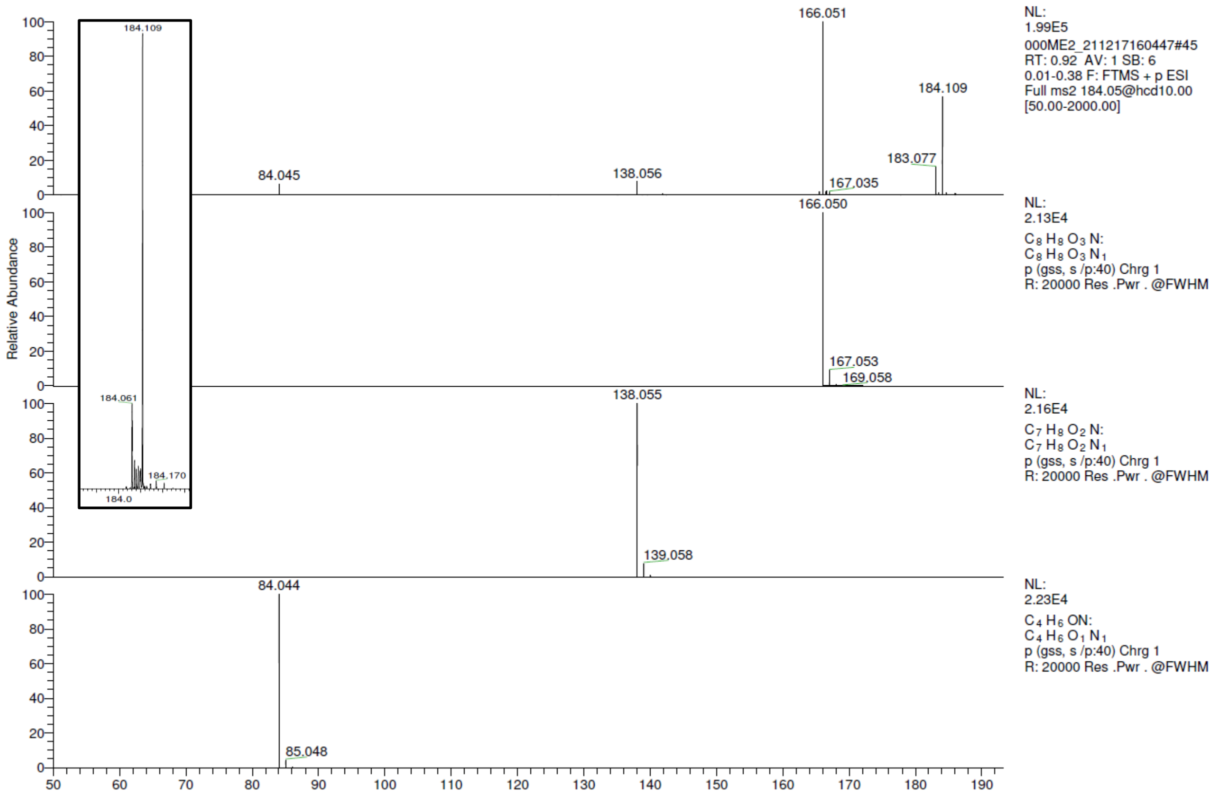

Figure S4. Collision-induced dissociation (CID) high resolution mass spectrum of mass-selected protonated hematinic acid showing its fragmentation pattern starting with consecutive losses of H_2O and CO, calculated spectra for the fragments below for comparison. The inset on the top left shows a zoom into the mass-selected region in the measured CID spectrum. Two signals with *m/z* 184 were present in the original spectrum (see Figure S3 top row), most of the molecular ion of hematinic acid (*m/z* 184.061) was fragmented by CID.
